# Decomposing the rural-urban gap in the factors of under-five mortality in sub-Saharan Africa? Evidence from 35 countries

**DOI:** 10.1186/s12889-019-6940-9

**Published:** 2019-05-21

**Authors:** Sanni Yaya, Olalekan A. Uthman, Friday Okonofua, Ghose Bishwajit

**Affiliations:** 1 School of International Development and Global Studies, University of Ottawa, 120, University Private, Ottawa, ON Canada; 2 Warwick Centre for Applied Health Research and Delivery (WCAHRD), Division of Health Sciences, Warwick Medical School, University of Warwick, Coventry, CV4 7AL UK; 3 Women’s Health and Action Research Centre, Benin City, Nigeria; 4 University of Medical Sciences, Ondo City, Ondo State Nigeria; 5 Centre of Excellence in Reproductive Health Innovation (CERHI), University of Benin, Benin City, Nigeria

**Keywords:** Under-5 mortality, Global Health, Sub-Saharan Africa, Decomposition, Urban-rural

## Abstract

**Background:**

Understanding urban-rural gap in childhood survival is essential for health care interventions and to explain disparities in the determinants of Under-5 mortality. There is dearth of information about the factors explaining differentials in urban-rural Under-5 mortality especially in sub-Saharan Africa (SSA). In this study, we sought to quantify the contributions of bio-demographic, socioeconomic and proximate factors in explaining the urban-rural gap in Under-5 mortality in SSA.

**Methods:**

This study utilized secondary data from Demographic and Health Survey (DHS) in 35 sub-Saharan countries conducted between 2006 and 2016. Child (aged 0 and 59 months) death was the outcome variable in this study. Oaxaca-Blinder decomposition was used to decipher urban-rural gap in the factors of Under-5 mortality.

**Results:**

Significant urban-rural differentials were observed in Under-5 mortality across bio-demographic, socioeconomic and proximate factors. In the decomposition model, about 44.27% of urban group and 74.71% of rural group had Under-5 mortality in sub-Saharan countries. Maternal age, education, use of newspaper, TV, wealth index, total children ever born, size of baby and age at first birth contributed towards explaining urban-rural gap inUnder-5 mortality.

**Conclusion:**

These findings could be contributory to health care system improvement and socioeconomic developmental plans to address under-5 mortality in SSA. Strengthening maternal and child health (MCH) programmes, specifically in rural areas and improving health care services would help to ensure overall child survival.

## Background

Sustainable Development Goal-3 (SDG-3) focuses on ensuring healthy lives and promoting the well-being for all, with specific targets to end preventable deaths of neonatal, infant and Under-5 by 2030 and to achieve universal health coverage (UHC), through access to quality, safe, effective, affordable and essential health care services [1]. This global effort is geared towards attaining substantial progress in increasing life expectancy with reduction in widespread diseases associated with early mortalities [1]. Over the past decades, childhood and maternal deaths have become a priority issue in public health. Global interventions have brought a remarkable decline of Under-5 mortality rates (U5MR) [2]. In 2015, global U5MR was 43 per 1000 live births, which was approximately half of 91 deaths per 1000 live births in 1990 [2]. Notably, the Millennium Development Goal 4 (MDG-4) targeted at childhood mortality reduction remained largely unmet in several countries in SSA.

Globally, about 6.3 million Under-5 death was reported in 2013 compared with12.7 million deaths in 1990 [3]. In 2015, about 5.9 million Under-5 children died including 45% neonatal deaths [4]. Quite remarkably, about half of the deaths occurred in SSA region alone [4]. In spite of the overwhelming estimates, there has been significant decline in childhood mortalities at global stage [3–5].

Under-5 mortality is regarded as a prominent indicator of the progress of societal value system in health care management. Effective prevention and control of childhood diseases, improved health care programmes, such as immunization and provision of vitamin supplementation are regarded as key factors in the decline of Under-5 death [6]. Effort to improve childhood survival has been persistent without prejudice to the background of beneficiaries, particularly where they live and their economic status.

The disparities in access and utilization of health care services have hampered the target of promoting Universal Health Coverage (UHC), including widespread reduction in childhood mortalities. Differentials in geographical area of residence and socioeconomic inequalities have been associated with several health indices [7, 8]. Compared to the disadvantaged, wealthier individuals enjoy greater access to quality health care services, while in most countries the rural dwellers and people of low socioeconomic class have been deprived in terms of accessibility to health care services [7–9]. The vulnerable population and those living in areas with difficult terrains, as a result of their economic and social exclusion, face hindrances in accessing health care services or participating in treatment or preventive interventions and consequently be at higher risk of early death. The reasons for higher rate of under-utilization of health care services among the disadvantaged communities have been attributed to affordability, lack of awareness, long distances to health facilities, social norms and discrimination [9].

In SSA, significant differences in childhood mortalities across several individual, household and community-level factors have been reported [10–13]. The situation is considerably worse for the under-privileged communities when it comes to health care seeking behavior and the quality of the services they receive. In many resource-constrained settings, interventions have been targeted to address the inequities and inequalities in under-5 mortalities. The focus has majorly been on the associations between individual-level factors and childhood mortality, which has not been investigated rigorously at national level [14].

There is paucity of data on Under-5 mortality, specifically the decomposition of mortality by urban-rural differences, which limits the understanding of the depth of the problem for evidence-based interventions. In the light of the above, we sought to examine the rural-urban differences in Under-5 death in SSA. Furthermore, it is paramount to generate discussion on childhood mortality and to enhance knowledge of the issues associated with childhood deaths. Results from this study would help policy and decision makers to effectively plan resources in regions with high U5MR.

## Methods

### Data source

Data used in this study were collected from Demographic and Health surveys in the following countries: Angola, Benin, Burkina-Faso, Burundi, Cameroon, Chad, Comoros, Congo, Cote d’Ivoire, Democratic Republic of Congo, Ethiopia, Gabon, Gambia, Ghana, Guinea, Kenya, Lesotho, Liberia, Madagascar, Malawi, Mali, Mozambique, Namibia, Niger, Nigeria, Rwanda, Sao Tome & Principe, Senegal, Sierra Leone, Swaziland, Tanzania, Togo, Uganda, Zambia and Zimbabwe. In this study, we used data from entire populations to compare Under-5 mortality across several characteristics for the surveys undertaken at various period of time [15]. Only the latest data on children born in the five years prior to the interview were used in the analysis. DHS surveys use multistage cluster sampling methods for data collection. The surveys are conducted by trained interviewers using structured questionnaires. A major component of the questionnaire for women’s survey is maternity history where women are asked about their birth histories including parity and child mortality. Data on birth history are also available as separate dataset for individual children aged 0–59 months that include information on date of birth, sex of the child, current age, age at death (for dead children), and relevant background characteristics. Table 1 shows details of the selected countries.

**Table 1:** Pooled Demographic and Health Surveys (DHS) data from 35 sub-Saharan countries, 2006–2016

Country	Year	Sample size
Central sub-Saharan		
Sao Tome & Principe	2008/09	1931
Angola	2015/16	14,322
Congo	2011/12	9329
Gabon	2012	6067
Democratic Republic Congo	2013/14	18,716
Chad	2014/15	18,623
Cameroon	2011	11,732
Eastern sub-Saharan		
Burundi	2010	7742
Madagascar	2008/09	12,448
Comoros	2012	3149
Ethiopia	2016	10,641
Kenya	2014	20,964
Malawi	2015/16	17,286
Mozambique	2011	11,102
Rwanda	2014/15	7856
Tanzania	2015/16	10,233
Uganda	2011	7878
Zambia	2013/14	13,457
Zimbabwe	2015	6132
Southern sub-Saharan		
Lesotho	2014	3138
Swaziland	2006/07	2812
Namibia	2013	5046
Western sub-Saharan		
Nigeria	2013	31,482
Guinea	2012	7039
Niger	2012	12,558
Benin	2012	13,407
Ghana	2014	5884
Burkina-Faso	2010	15,044
Cote d’Ivoire	2011/12	7776
Liberia	2013	7606
Mali	2012/13	10,326
Senegal	2010/11	12,326
Sierra Leone	2013	11,938
Togo	2013/14	6979
Gambia	2013	8088

### Measurement of variables

#### Outcome variable

The primary outcome variable of this study was under-5 death occurring in the 5 years preceding data collection. Data on under-5 death were collected by mother’s recall.

#### Explanatory variables

The systematic conceptual framework by Moseley [16] was the basis of selecting the explanatory variables in this study. Here, we identified prominent factors of Under-5 mortality as available in DHS datasets. The place of residence: urban vs rural; maternal age groups; 15–19, 20–24, 25–29, 30–34, 35–39, 40–44 and 45–49 years; maternal and paternal education: no education, primary, secondary and higher; sex of household head: Male vs female; read newspaper/magazine, use of radio & watch TV: not at all, less than once a week, at least once a week, almost every day; number of children ever born: 1–4 vs > 4; marital status: not currently married vs currently married/living with partners; employment status: not working vs currently working; birth order: 1st – 3rd, 4th – 6th, > 6th; type of birth: singleton vs multiple; sex of child: male vs female; size of child: large, average, small; maternal age at first birth: < 22 years, 22–28, > 28 years. Wealth index is calculated by using principal components analysis (PCA) that involves assigning scores on the indicator variables such as; floor type, wall, roof, water source, sanitation facilities, radio, electricity, television, refrigerator, etc. Following that, the factor coefficient scores (factor loadings) and z-scores were calculated. Finally, for each household, the indicator values were multiplied by the loadings to produce the household’s wealth scores. The standardized score was used to categorize the overall assigned scores to poorest, poorer, middle, richer, richest levels.

### Ethical clearance

We conducted the analyses using publicly available data from demographic health surveys. Prior to each interview, participants gave informed consent to participate in the survey. DHS Program is consistent with the standards for ensuring the protection of respondents’ privacy. ICF International ensures that the survey complies with the U.S. Department of Health and Human Services regulations for the respect of the right of human subjects. No further approval was required for this study since the data is secondary and available in the public domain. More details about data and ethical standards are available at: http://goo.gl/ny8T6X.

### Multicollinearity testing

Correlation matrix was used in collinearity diagnostic approach to omit some of the correlated variables to reduce multicollinearity. Examining the correlation matrix is helpful to detect multicollinearity using a cut-off of 0.6 known to cause concern in multicollinearity [17]. Due to significant collinearity with birth order, total children ever born (r = 0.814) and maternal age (r = 0.684) were removed from the model, as “birth order” was adjudged more vital to investigate under-five mortality. Further, maternal education had dependence with paternal education (r = 0.646). Therefore, maternal age, total children ever born and paternal education were therefore removed following multicollinearity testing.

### Data analysis plan

There were no missing values for child survival status (whether a child was dead or alive). However, dead children with missing age at death were excluded from the analysis, which accounted for 0.2% of the cases. Overall distribution of Under-5 mortality rate was calculated using summary statistics and Chi-square test.

We calculated the risk difference in under-five mortality between children from rural and urban areas. A risk difference greater than 0 suggests that under-five mortality is prevalent among children from rural areas (pro-rural inequality). Conversely, a negative risk difference indicates that under-five mortality is prevalent among children from urban areas (pro-urban inequality). Finally, we used logistic regression method to conduct the Blinder-Oaxaca decomposition analysis [18, 19, 20]. This method allows quantifying the gap between the “advantaged” and the “disadvantaged” groups.

The DHS stratification and the unequal sampling weights as well as household clustering effects were taken into account in the analysis to correct standard errors. Data analysis was carried out using STATA 14 (Statacorp, College Station, Texas, United States of America).

## Results

Overall Under-5 mortality varied by urban-rural place of residence was presented in Table 2. Mantel Haenszel test of homogeneity of odds ratio was used to test statistical significance using urban-rural as effect modifier. Mothers at extreme age intervals; 15–19, 40–44 & 45–49 had overall higher Under-5 mortality rate. For maternal education, births of women with no education had highest Under-5 mortality rate (78.1 and 79.3% respectively). Maternal media use (newspaper/magazine, radio and TV) had lower 70.0% Under-5 mortality rate. There were disparities in Under-5 mortality rate across the levels of other variables. Whereas, singleton births had 61.8%, multiple births had 21.40% Under-5 mortality rates. Male and female children reported 72.8 and 61.4 Under-5 mortality rate respectively. Large, average and small size of children reported 58.9, 60.0 and 94.0% Under-5 mortality rate respectively. Results showed statistically significant association of selected factors with Under-5 mortality.

**Table 2:** Distribution of Under-5 mortality in sub-Saharan Africa region

Variable	Number of children	Overall U5M per 1000 live births	Under-five mortality per 1000 live births		*P*-value
			Urban (57.8)	Rural (71.2)	
Maternal age					
15–19	24,096	76.0	67.9	79.3	0.000*
20–24	84,386	67.5	61.0	70.5	
25–29	100,807	62.6	52.6	67.2	
30–34	76,274	62.5	53.4	66.7	
35–39	53,054	68.9	59.2	72.8	
40–44	24,743	76.1	64.4	80.1	
45–49	7697	100.0	92.6	102.0	
Maternal education					0.000*
No education	157,975	78.1	67.6	80.4	
Primary	127,208	64.1	63.4	64.4	
Secondary	76,864	53.7	50.8	57.1	
Higher	8962	33.7	32.7	37.2	
Paternal education					0.000*
No education	125,129	79.3	67.4	81.7	
Primary	93,217	65.9	63.3	66.6	
Secondary	86,979	60.1	53.3	65.7	
Higher	17,969	47.4	44.5	53.8	
Sex of household head					0.000*
Male	296,359	68.3	57.4	72.6	
Female	74,698	62.8	59.3	64.8	
Mother read newspaper/magazine					0.000*
Not at all	314,133	70.0	61.2	73.0	
Less than once a week	31,077	52.6	50.3	54.9	
At least once a week	22,331	49.3	47.0	53.0	
Almost every day	2758	54.4	54.0	55.1	
Mother listen to radio					0.000*
Not at all	157,566	71.3	60.7	74.3	
Less than once a week	68,953	67.0	58.8	70.6	
At least once a week	121,064	62.7	55.5	67.2	
Almost every day	23,036	62.4	56.6	66.5	
Mother watch TV					0.000*
Not at all	242,275	72.3	66.4	73.4	
Less than once a week	41,082	64.8	61.4	66.9	
At least once a week	66,085	54.6	51.8	59.5	
Almost every day	20,859	50.8	49.8	54.1	
Household wealth index					0.000*
Poorest	94,616	73.2	61.7	74.1	
Poorer	81,513	72.9	68.4	73.5	
Middle	72,629	67.8	61.4	69.8	
Richer	65,475	63.9	61.5	65.9	
Richest	56,824	51.9	51.5	54.0	
Total children ever born					0.000*
1–4	236,662	58.4	52.0	61.7	
> 4	134,395	82.7	74.2	85.1	
Marital status					0.000*
Not currently married	46,303	69.3	70.0	68.9	
Currently married/living with partner	324,745	66.9	55.4	71.4	
Employment status					0.000*
Unemployed	137,150	65.2	55.2	69.9	
Employed	222,255	69.6	60.3	73.3	
Birth order					0.000*
1–3	206,773	62.8	54.2	67.5	
4–6	114,302	65.4	60.0	67.3	
> 6	49,982	89.2	79.5	91.4	
Type of birth					0.000*
Singleton	357,927	61.8	53.1	65.5	
Multiple	13,130	214.0	181.2	228.7	
Sex of child					0.000*
Male	187,813	72.8	63.1	77.0	
Female	183,244	61.4	52.4	65.2	
Size of child					0.000*
Large	132,784	58.9	49.7	63.1	
Average	159,225	60.0	48.0	65.0	
Small	59,871	94.0	89.1	95.7	
Maternal age at first birth					0.000*
< 22	268,499	68.1	59.6	71.4	
22–28	69,021	59.0	49.8	64.7	
> 28	7647	63.7	48.0	77.6	

*Significant at 0.05

*U5M* under-five mortality

### Magnitude and variations in rural-urban inequality in under-five mortality

**Fig. 1:**
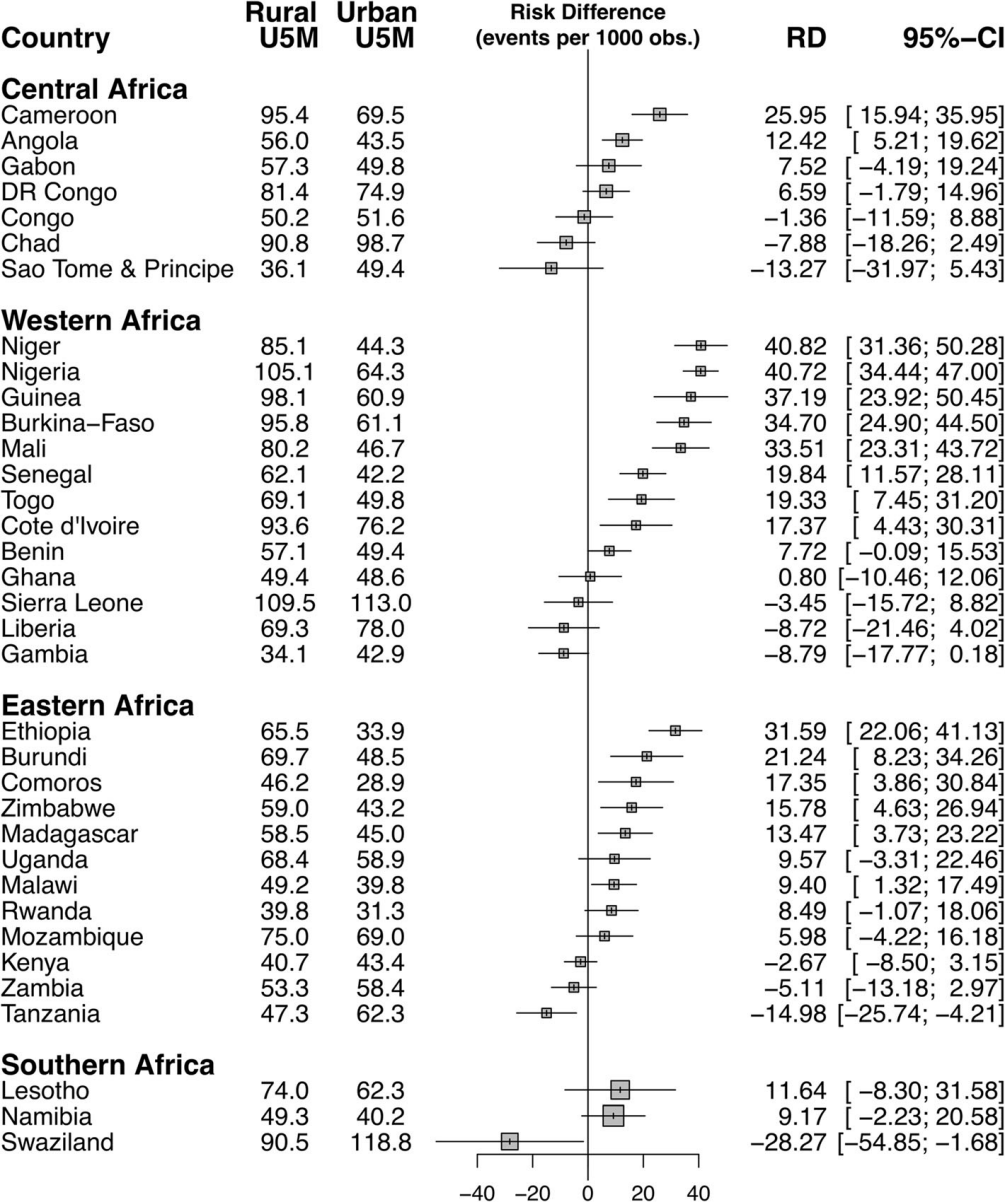
Risk difference between children from rural and urban areas in under five mortality rates by countries

**Fig. 2:**
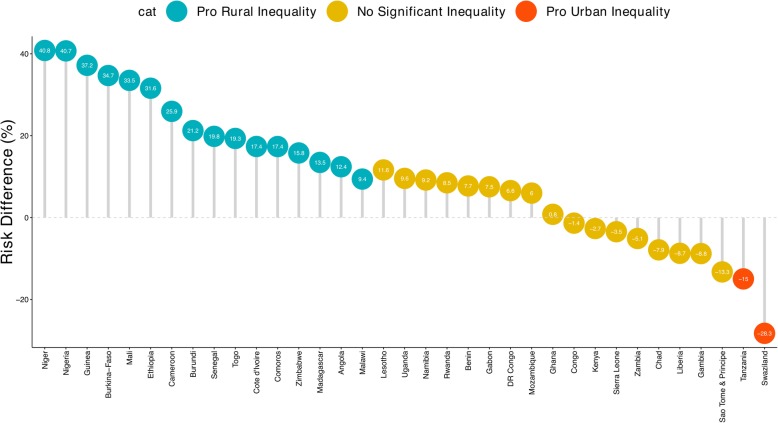
Risk difference between children from rural and urban areas in under five mortality rates by countries

Figures 1 and 2 show the risk difference (measure of inequality) between rural and urban areas across the 35 countries studied. Out of the 35 countries included in this analysis, 16 countries showed statistically significant pro-rural inequality (i.e. under-five mortality more prevalent among children from rural areas), 2 showed statistically significant pro-urban inequality (i.e. under-five mortality more prevalent among children from urban areas) and remaining 17 countries showed no statistically significant inequality. As illustrated by Fig. 1, in Central Africa the urban-rural difference was largest for Cameroon (25.95) and lowest for Congo (− 1.36). In Western Africa, the urban-rural difference was largest for Niger (40.82) and lowest for Ghana (0.8). In Eastern Africa, the difference was largest for Ethiopia (31.59) and lowest for Kenya (2.67). Among the three countries Southern Africa, Swaziland had the largest urban-rural difference (− 28.27) and Namibia had the lowest (9.17). In the pooled analysis, Niger still had the highest pro-rural inequality and followed by Nigeria (40.7) and Guinea (37.2), while Tanzania (15) and Swaziland (28.3) were the only countries to show pro-urban inequality (Fig. 2). Two of the seven countries in Middle Africa showed statistically significant pro-rural inequality. In Western Africa, 8 of the 13 countries showed statistically significant pro-rural inequality. Similarly, in Eastern Africa, 6 of the 12 countries showed statistically significant pro-rural inequality. While in Southern Africa none of the three countries showed statistically significant pro-rural inequality. Tanzania and Swaziland were the only countries that showed statistically significant pro-urban inequality.

### Relationship between under-five mortality rate and magnitude of inequality

**Fig. 3:**
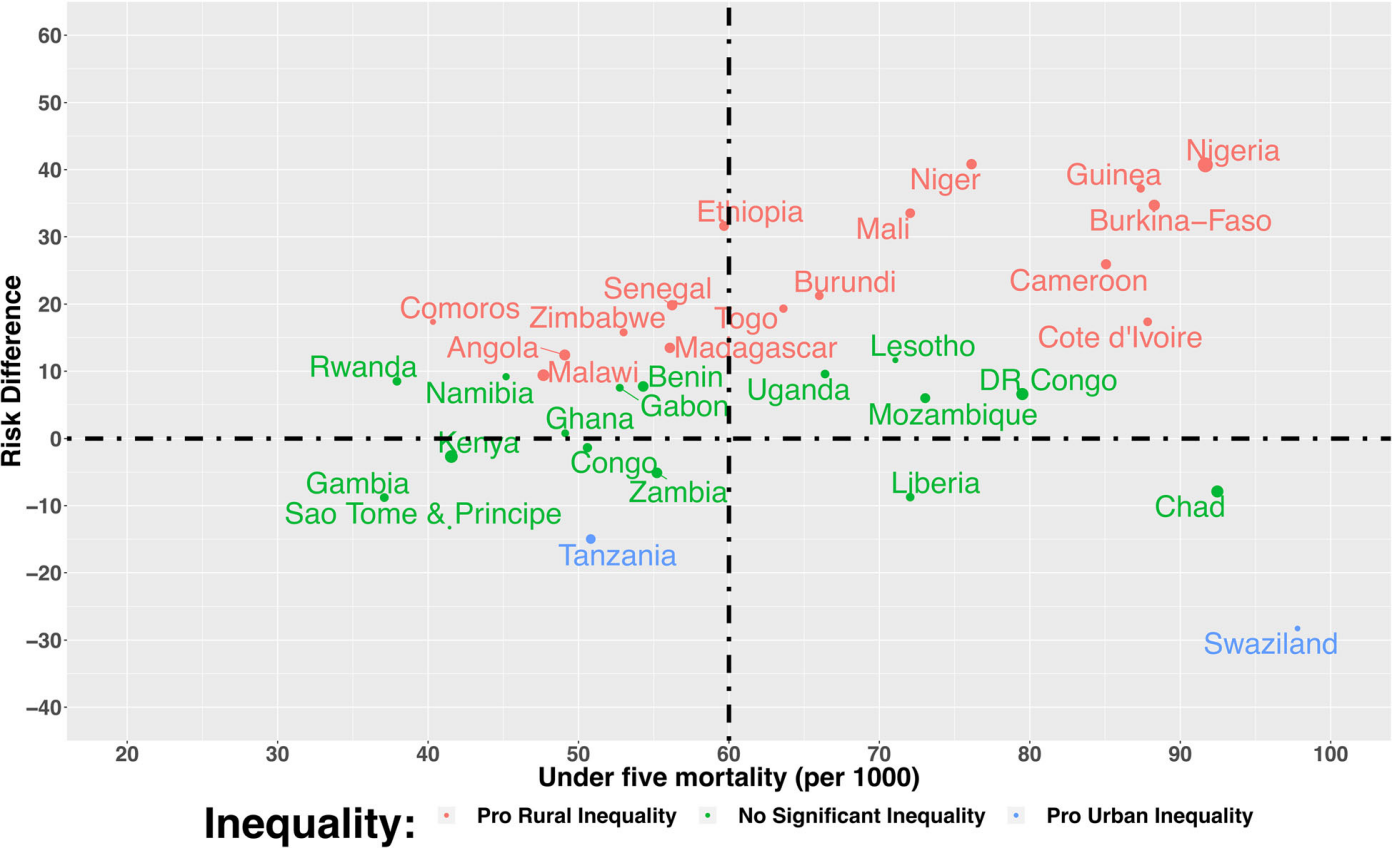
Scatter plot of rate of under-five mortality and risk difference between children from rural and urban areas in under five mortality rates by countries

Figure 3 plot the relationship between under-five mortality rate and magnitude of inequality for all the 35 countries. We grouped countries into 4 distinct categories:

1. high under-five mortality and high pro-rural inequality such as Nigeria
2. high under-five mortality and high pro-urban inequality such as Swaziland
3. low under-five mortality and high pro-rural inequality such as Comoros
4. low under-five mortality and high pro-urban inequality such as Tanzania

### Decomposition of rural-urban inequality in under-five mortality

**Fig. 4:**
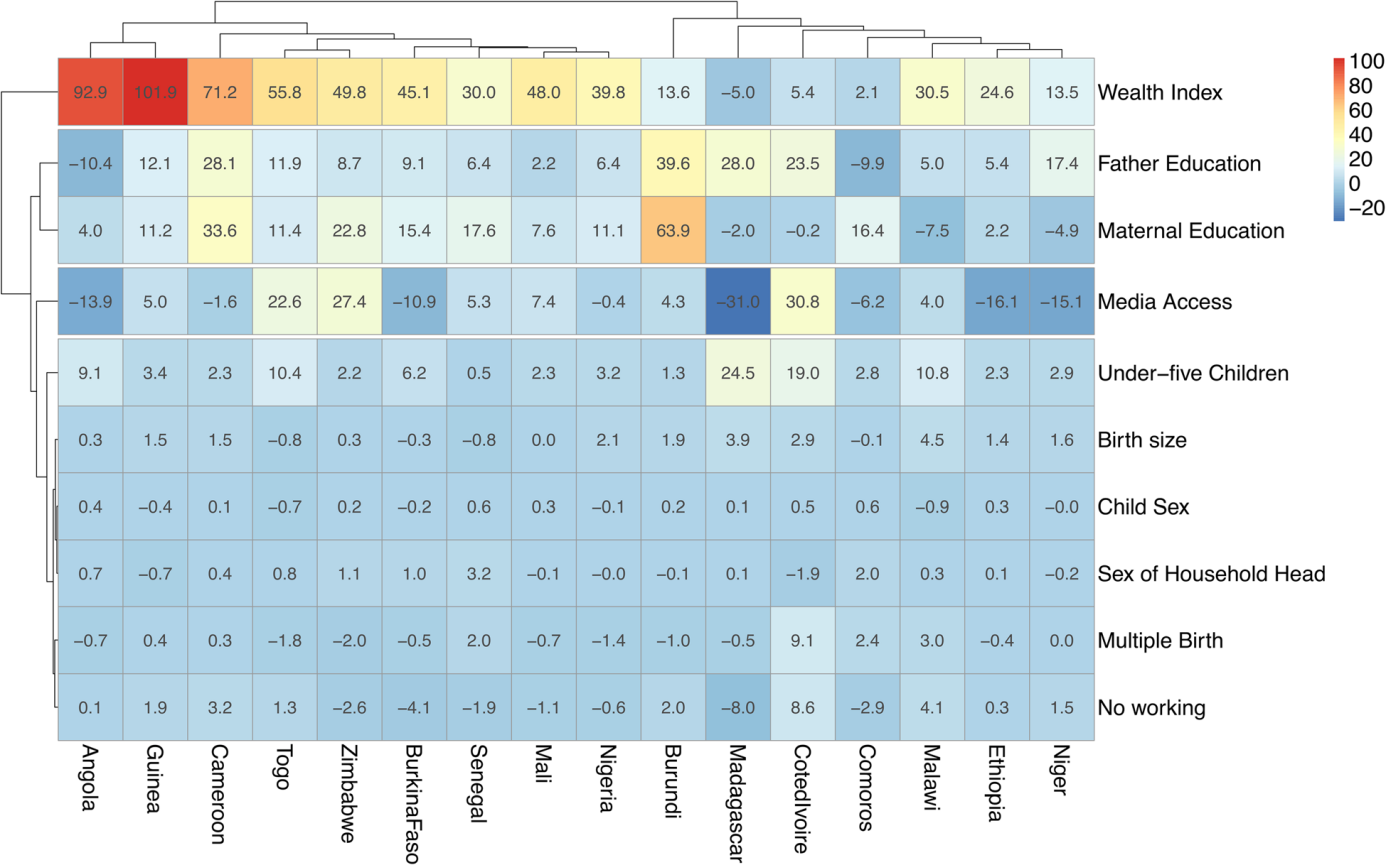
Contributions of differences in the distribution ‘compositional effect’ of the determinants of under-five mortality to the total gap between children from rural and urban areas in under five mortality rates by countries

Figure 4 shows the detailed decomposition of the part of the inequality that was caused by compositional effects of the determinants. The important factors responsible for the inequality varied across the countries. On average, wealth index and media access were the most important factor in most countries. In Niger, the largest contributions to the rural-urban inequality in under-five mortality was frequency of watching television, followed by maternal education attainment and wealth index. However, partner / husband’s education attainment was narrowing the inequality in under-five mortality between children from rural and urban areas. Notably, in Madagascar media access had the largest contribution the urban-rural inequality followed by father’s education and presence of under-five children. Child sex, household head’s sex, parity, and mother’s employment status didn’t show any significant contribution to the inequality in any of the countries.

## Discussion

In this study, we identified the pattern of Under-5 children mortality using pooled data from 35 sub-Saharan Africa countries. In addition, we obtained the urban-rural gap across various determinants of Under-5 mortality. Similar to previous reports, this study found a range of factors associated with the death of under-five children in developing countries [6, 10, 20, 21]. We also found noticeable inter-country differences in the risk-difference in under-five mortality between urban and rural areas. In a great majority of the countries, the prevalence of mortality was higher in the rural areas, with the only exception to this being Tanzania and Swaziland. In terms of region, the greatest pro-rural inequality was observed for Western Africa (in Niger) and the greatest pro-urban inequality was observed for Southern Africa (in Swaziland). Inequalities of this alarming magnitude suggests a chronic underestimation of the child health issues in the rural areas and highlights a need for urgent intervention. The countries that showed low yet significant pro-rural inequality were Angola, Cote D’Ivoire, Madagascar. We didn’t find any significant pro-rural inequality in Southern Africa, however, there was a sizeable pro-urban inequality in Swaziland which is an interesting finding and require further investigation. Countries that are experiencing significant urban-rural inequalities should learn from the others in the region, such as Ghana and Kenya, and take urgent steps to ensure that progress is being made in line with the SDG targets.

Several factors explained Under-5 mortality including rural residence which accounted for higher risk of Under-5 death compared to urban residence. This could be supported by the fact that urban dwellers have higher likelihood to utilize health care services or could have better access to health care facilities; hence childhood illnesses could receive timely and appropriate treatment. Notably, very young age at first birth, children of higher birth order, multiple births and those with small size at birth had higher risk of Under-5 mortality. On the contrary, children from richest households, those from female headed households, births from educated women, maternal reading newspaper or magazine, watching television, children born to women in a marital union have reduction in the risk of Under-5 death than those born in a union and female child have lower risk of Under-5 mortality. These findings are consistent with previous reports [6, 10, 22–26]. Educated mothers are more empowered to make decisions on the use of health care services and adequate utilization of preventive and therapeutic health services which have higher impact on children survival. Moreover, marital protection may be peculiar to the Under-5 period in sub-Saharan Africa context because culturally, babies and their mothers enjoy a lot of social supports and other familial in the first few months after birth. Examining higher risk of Under-5 death among births from maternal young age at first birth, this presupposes that physical and physiological maternal maturity is reached for children survival.

In addition, the challenges of childhood survival explain the fact that low birth weight children are more likely to be susceptible to problems connected to preterm delivery. Higher risk of Under-5 death among mothers with very young age at first delivery shows that teenage pregnancy is unsafe for child survival. The lack of physical maturity needed for healthy pregnancy outcomes is a major issue that could be responsible for Under-5 death. Urban-rural gap related to socioeconomic and proximate factors of Under-5 mortality identified are crucial to the utilization of childhood health care services.

In the decomposition analysis, maternal age and education, use of media, wealth index, number of children ever born, working status, size of baby, and age at first birth contributed towards explaining urban-rural gap in Under-5 mortality. On the contrary, sex of household head, marital status, type of birth and sex of child contributed to widening this gap. These findings are similar to the ones from previous studies [27–31]. These results can serve as important resources for designing children survival programs especially for effective health care intervention design.

Sub-Saharan African continent is considerably diverse with a multitude of ethnic, cultural and religious communities. In line with that, we found that the magnitude and directions of the associations explaining the rural-urban mortality varied widely across the countries studied. Thus, multifaceted geographically specific intervention may prove to be a potentially better approach for addressing the rural-urban differential in under-five mortality in sub-Saharan Africa with policies tailored to country-specific conditions.

### Strengths and limitations

The major strength of this study is that it utilized nationally representative data and the findings are generalizable for the Under-5 in sub-Saharan Africa countries. Nonetheless, a major drawback is the nature of cross-sectional study design, which is unable to sufficiently establish causality. Also, mortalities were measured based on self-reported data which can blur accuracy of results. For example, some Under-5 deaths were reported to have occurred within first 24 h. Perhaps, some of these might have been stillbirths leading to over-reporting. Underreporting of child deaths is always a concern when collecting birth histories from mothers. The mother may not wish to report such unfortunate events. Since the study utilized secondary data, variables on fertility behavior and child care practices which are mostly influenced by cultural norms, values and beliefs which could have affected the associations were not possible to analyze.

## Conclusions

This study identified that high Under-5 mortality rates persist in sub-Saharan Africa countries with significant urban-rural differences. Here, Under-5 mortality was explained by community-level factor, household factors and individual related factors. Based on these factors, maternal education and education of female child remain crucial approach to prevent child marriage or prolong the age at first birth. Maternal, newborn and child health care services will also improve the progress in Under-5 survival. The corollary of these findings is that sub-Saharan Africa children need better health-care support to reach the global target of reducing childhood deaths including neonatal, infant and under-five. To address the urban-rural discrepancies in childhood health outcomes, it is important not only to introduce a health and social protection scheme, but also to ensure equality and equity in those child welfare schemes.

## Abbreviations

DHS: Demographic health survey
IRB: Institutional review board
SDGs: Sustainable development goals
SSA: Sub-Saharan Africa
UHC: Universal health coverage
UMR: Under-five mortality
